# The Value of Large Sections in Surgical Pathology

**DOI:** 10.1155/2012/785947

**Published:** 2012-11-21

**Authors:** Maria P. Foschini, Chiara Baldovini, Yuko Ishikawa, Vincenzo Eusebi

**Affiliations:** Department of Biomedical and Neuro Motor Sciences, Section of Anatomic Pathology, University of Bologna, “M. Malpighi”, at Bellaria Hospital, Via Altura 3, 40139 Bologna, Italy

## Abstract

Large format sections (LS) first have been introduced in breast pathology more than a century ago. Since then, they constituted for longtime a research tool to better understand breast microanatomy and the relationship between radiological images and pathological features. Similarly LS have been used to study neoplastic, inflammatory, and degenerative diseases affecting various organs, as brain, lung, gastrointentinal tract, bone, urinary tract, prostate, and placenta. Currently LS are mostly applied to diagnostic routine to better stage tumours such as prostate and breast carcinomas or to correlate radiologic imaging to gross specimens. The purpose of the present paper is to review the historical background and the basis of the applications of LS in surgical pathology, with special emphasis on breast tumours.

## 1. Introduction

Large format sections (synonym macrosections) (LS) comprise an entire histological section of the organ under study. LS allow the histological study of a large part of the organ of interest, that include not only the lesions but also the surrounding tissues possibly inclusive of the entire margins at least along a plane of sectioning. The purpose of the present paper is to review the history and the present practical diagnostic applications of LS.

## 2. Historical Background

LS have been introduced in surgical pathology more than a century ago. The first study based on LS was published in 1906 by Cheatle [1] who realized that most patients were diagnosed to be affected by breast cancer only when the disease was too advanced to be cured. Therefore, as breast was the first organ to be studied, which has generated a large number of papers, this paper will first deal with breast tumours, followed by the application of LS in other organs.

 Dr. Cheatle used LS in cases of breasts affected by cancer to better understand the relation between the neoplastic mass and the surrounding normal tissue and the possible existence of premalignant changes [1–3].

The relation between breast cancer and the surrounding tissue was the object of a further study in 1939 by Ingleby and Holly [4]. Subsequently, Marcum and Wellings [5] improved and simplified the method which then was applied to study the early phases of breast cancer development [6, 7]. In 1973, Wellings and Jensen [8] analysed cases of in situ and invasive ductal and lobular in situ and invasive carcinoma leading to the proposal that most breast carcinomas arise in the terminal ductular-lobular unit (TDLU), in spite of the different morphological features.

These papers were paralleled with useful studies on mouse and human breasts to better understand the breast microanatomy [9–11] which was also accurately described by Going and Moffat [12] together with the distribution of the mammary lobes. Tot [13] based his theory of the sick lobe on studies performed on LS.

Although LS, especially at the beginning, were used in a few laboratories only, their impact on the evaluation of multifocality and multicentricity of breast cancer appeared relevant. Egan [14], in a seminal paper, demonstrated that the incidence of cases showing multifocal breast cancer was more frequent than that obtained from studies based on conventional small block slides. This led to the discovery that multifocal breast carcinomas have a worse prognosis in comparison with unifocal lesions. Similar results were subsequently obtained in 1986 by Sarnelli and Squartini [15] and, more recently, by Tot et al. [16, 17].

Since 1992, Faverly et al. [18] employed LS for a three-dimensional reconstruction of the breast glandular tree, in order to evaluate the extention and type of growth of different types of in situ duct carcinomas (DCIS) [19, 20]. These studies greatly increased the knowledge on the extent and distribution of DCIS, as they demonstrated that DCIS is frequently a multifocal process and that multifocality is a typical feature of low grade rather than high grade DCIS. Along this line were the results obtained on multifocality and multicentricity by Tot et al. [16, 17] and Foschini et al. [21].

In the last decade, LS were demonstrated to be useful to correlate radiological findings and pathology as it is simple to compare radiological images to the large histological sections [22]. Specifically the widespread use of mammographic screening for early detection of breast cancer identifies numerous benign lesions that can be difficult to interpret on the mammogram. This was mostly done by Tot et al. [23] who gave a better definition of several benign breast lesions, among which radial scar, which does not need any further diagnostic investigation. More recently Tot and Tabár [24] correlated the nuclear magnetic resonance (NMR) findings with LS demonstrating that LS can help to better diagnose the different histological lesions and features that characterize all the breast malignant tumours.

## 3. Methods 

In spite of their utility in diagnostic pathology, LS are still used in a relatively few laboratories, mainly as a consequence of the fact that their preparation is perceived as more timeconsuming and expensive than conventional blocks. This difference is only apparent as in LS a large portion of tissue is examined that is far superior to the conventional small blocks. Tot [25] calculated the costs of LS in daily practice and compared them with those obtained from conventional blocks, demonstrating that LS costs do not substantially differ from those of accurate conventional blocking. Tucker [26] calculated the cost of LS in a breast care centre and concluded that the LS costs increase from 6.02 USD in cases of lumpectomies to 18.58 USD in cases of mastectomies which was regarded relatively inexpensive and balanced by a better staging and more accurate evaluation of resection margins [26]. As a consequence additional surgical procedures were lowered in number, which led to a decrease of the overall cost of each single patient's treatment [26]. 

The method to obtain LS has been previously described in several papers [27], as well as it is described by Tucker [26] in the present issue.

At our institution, the breast specimens (as well as specimens from other organs) are sliced with a large blade, into sections 5 to 10 mm thick, possibly under radiological guidance or following indications given by the surgeon. One to three LS are obtained from each case and in addition the of rest of the surgical specimen is embedded using traditional small blocks. Additional automatic processor is used to work overnight. In cases of small quadrants, when the LS major axis is less than 5 cm, the entire specimen is embedded using the same procedure employed for prostatectomies as illustrated by the specific paper of this issue [28]. This last procedure is less time consuming with a shorter turn-around time to obtain the LS. In addition these “smaller” LS are easy to manage at the microscope and can be easily digitalized into virtual slides with the proper hardware (personal comunication). 

Paraffin blocks from paraffin embedded LS [26, 27, 29] are then cut with a dedicated macrotome. Finally haematoxylin-eosin large slide is obtained. Orientation is maintained during the whole embedding process and reported in the final slide. 

When immunohistochemistry or molecular studies are needed, areas of interest are selected from the LS and cut to obtain small conventional blocks [30].

LS can be used also for 3-dimensional reconstructions as previously shown [19, 29] and summarized as follows. 

LS blocks are deparaffinided by melting paraffin at 60°C from 3 to 4 hours, subsequently tissue is immersed in xylene (four times) for at least 24 h to remove residual paraffin. Tissues are then rehydrated as follows: 50% absolute alcohol and 50% xylene (1 hour), absolute alcohol (2 hours), and 70% alcohol (2 hours). Blocks are washed overnight in distilled water, stained in Harris' hematoxylin for 4-5 min, rinsed in tape water for 10 min, and immersed in four baths of acid alcohol for 8 min each. Finally tissues are dehydrated, through a graded series of alcohol to xylene, and finally immersed in methyl salicylate for one night. 3D examination is performed using a stereomicroscope. The H&E-stained LS slide is used to retrace the lesions to examine on the corresponding cleared tissues.

## 4. Large Sections in Breast Pathology

LS are useful during the everyday breast routine practice to better evaluate the tumour dimension, the in situ carcinoma extension, and the resection margins. In the series studied by Foster et al. [31], LS gave more information than conventional blocks in 172 cases out of 656, as they evidenced additional findings of potential clinical use, as involved margins, minute multiple foci of invasive or in situ carcinoma, or change in size and extent of the tumour under study. 

**Figure 1:**
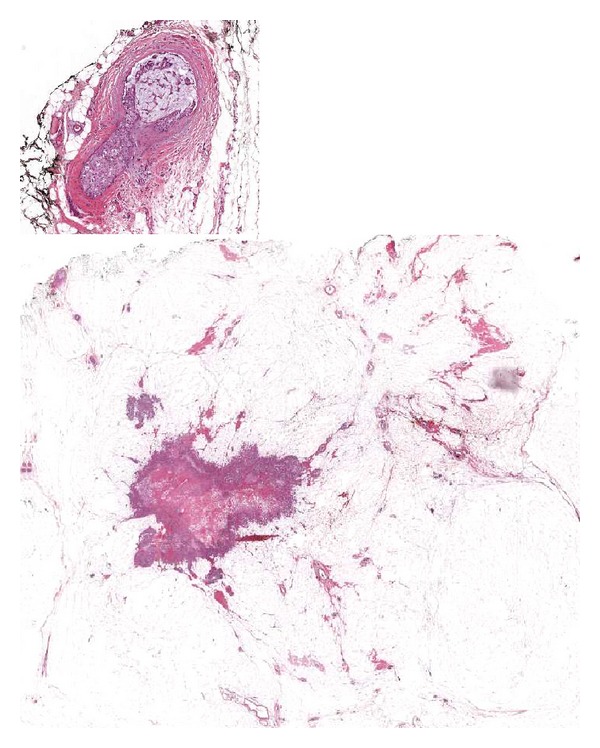
Quadrantectomy: a small focus of DCIS, immersed within the fatty tissue, is present in a surgical margin (inset).

Correct evaluation of resection margins has become an increasingly important issue especially in cases treated with quadrantectomy. LS are cut and oriented according to the radiological images and the indications given by the surgeon, and orientation is maintained during the paraffin embedding procedures. This allows the exact evaluation of the relationship between invasive or in situ carcinoma and the adjacent surgical margin. By contrast conventional blocking is based on gross inspection at naked eye of the lesions and on palpation of the tissues; therefore, minute cancer foci, immersed within the fatty breast stroma can escape from examination (Figure 1).

**Figure 2:**
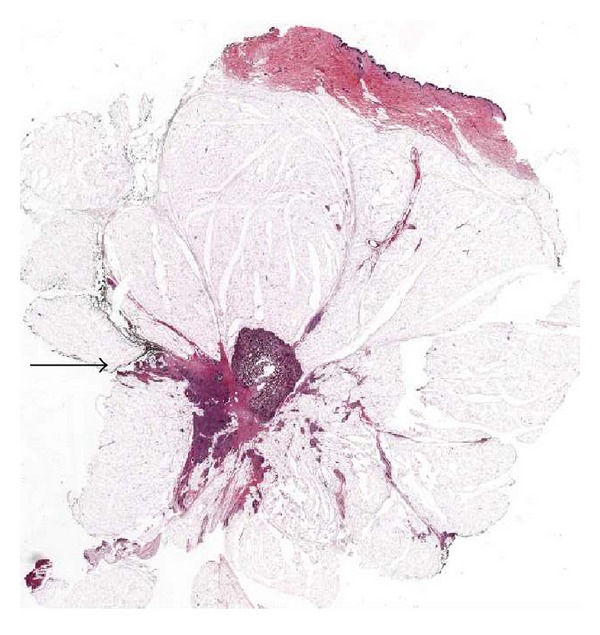
Quadrantectomy: the real inked margin can be easily recognized and distinguished from ink migration into fissures of the fat tissue (arrow).

In addition having the possibility to visualize the whole section of the breast specimen, it is easier to distinguish the real inked margin from ink migration through tissue fissures frequently present in breast tissues as also stated by Tucker [26] (Figure 2).

Exact evaluation of tumour dimension is at the basis of a correct staging. Jackson et al. [32] compared two series of breast carcinomas, one studied with conventional histological method and the second studied with LS. Accordingly the correct size of the tumour was assessed in all the cases studied with LS, while it was assessed in 63% only of the cases studied by conventional histology. We compared the tumour size on a series of 102 consecutive quadrantectomies evaluated with both LS and conventional blocks. Accordingly, in 9/102 (8,8%) LS helped to correctly assess the dimension of the tumour better than the small blocks, especially in invasive lobular carcinoma, where the macroscopic borders of the tumours were ill defined and difficult to be measured at macroscopical level only [33].

 In addition the widely spread breast cancer screening programs lead to the detection of a high rate of in situ carcinomas and of microscopic foci of invasive carcinomas.

Due to the use of LS, it is becoming evident that breast cancer often presents with multiple foci and unifocal, multifocal and multicentric in situ, or invasive carcinomas [16, 17, 21] appear better demonstrated. Tot et al. [17], on a study performed on 574 consecutive cases studied with LS, found that invasive carcinomas was multifocal in 24% and diffuse in 5% of the cases, percentages that are largely superior to those published in paper based on conventional blocking. 

The prognostic value of multifocality in breast cancer has been widely debated in the literature. Nevertheless, studies performed on LS [14, 17] have shown that multifocality has a great impact on survival, as the risk of death for breast cancer is higher in patients with multifocal and/or diffuse carcinomas when compared with those with unifocal carcinomas. Similar results were obtained at our institution, when LS were compared to conventional small blocks in a series of 102 consecutive cases diagnosed during the year 2010. The most consistent additional information that LS have provided, over conventional small blocks, was tumour multifocality (27/102 cases, 26,4%). Patients with multifocal tumours exhibited axillary lymph-node metastases in 71,42% while those with unifocal tumours showed axillary nodal involvement in 40,54% [34]. These data confirm that multifocality can be useful in the evaluation of the risk of axillary involvement by breast cancer metastases and confirm that the detection of multiple breast cancer foci has a great prognostic impact and therefore should be carefully searched in all cases of breast cancer.

**Figure 3:**
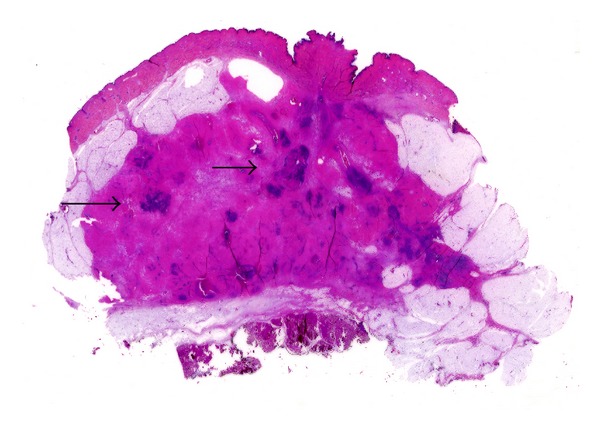
LS performed on a case treated with neoadjuvant chemotherapy evidence small foci of residual invasive breast carcinoma (arrows).

In cases of breast cancer diagnosed in advanced stages, surgery is preceded by neoadjuvant chemotherapy with the aim of reducing the tumour mass. In order to correctly stage breast cancers treated by neoadjuvant chemotherapy, it is of vital importance to evaluate the presence of residual tumour and the degree of tumour regression [35]. In cases showing good response to neoadjuvant chemotherapy the tumour mass greatly decreases and sometimes is difficult to evaluate on macroscopy. Therefore, when histology is performed on conventional blocks, small residual tumour foci frequently escape detection. To this purpose the use of LS improves the correct evaluation of the resection specimen (Figure 3). Embedding the whole area previously occupied by the tumour can improve the chance to detect even small residual neoplastic foci.

Finally LS are of use also in the evaluation of other prognostic parameters, such as vascular invasion, which is used to plan chemotherapy. Comparing conventional blocks and LS on 102 consecutive cases treated at this institution, in 14 cases (13,72%) vascular invasion was detected in LS only [34].

## 5. Large Section in Anatomic and Surgical Pathology

Similarly to breast pathology, during the last century, LS have been used to shed light on different pathological processes of various organs.

**Figure 4:**
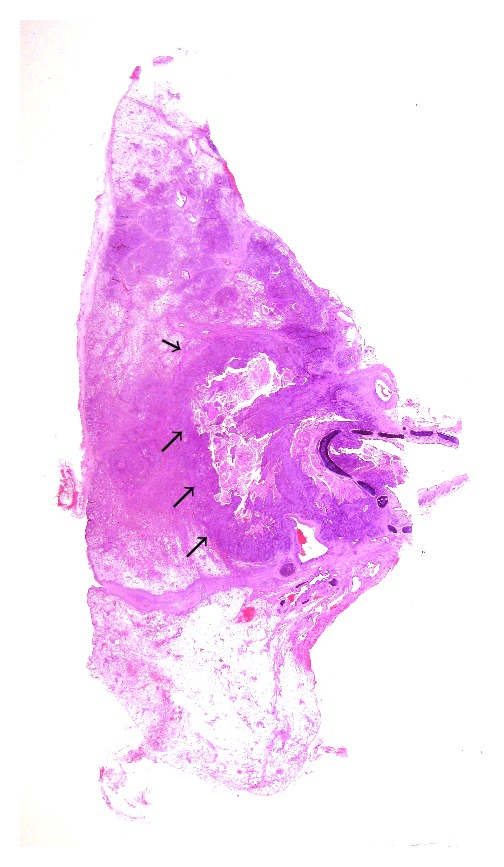
LS clearly visualizes the relationship between lung cancer (indicated by the arrows) and the surrounding obstructive pneumonia.

**Figure 5:**
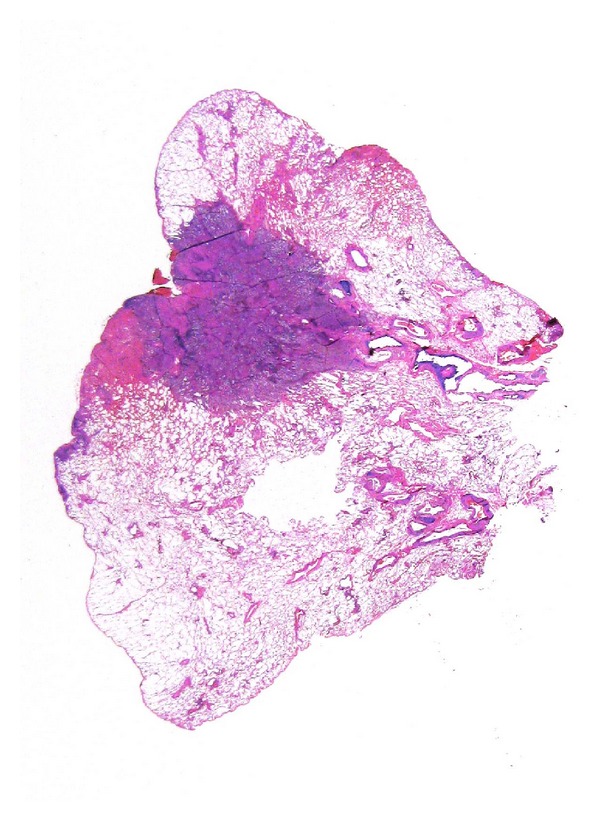
Lung cancer is easily staged using LS that evidence pleural invasion and distance from the bronchial margin.

Since 1960 LS have been proven to be useful to study the extension and distribution of lung diseases as emphysema [36] and, more recently the size of the tumours, to plan radiotherapy in cases of nonsmall lung cancer [37]. In current practice, LS are useful in staging cases of lung cancer (Figure 4) as the relationship between the tumour mass and the adjacent obstructive pneumonia is difficult to establish at macroscopic level (Figure 5). In addition the assessment of pleural invasion or resection margins that may be problematic on macroscopic examination, are readily evaluated on LS.

LS have been widely used in bone pathology to compare the radiological images to the different pathological aspects of benign and malignant bone tumours [38–40]. Specifically, type of growth and extension of osteosarcoma and chondrosarcoma were elucidated comparing radiological imaging and LS from surgical resection specimens. Bertoni et al., [41] using LS, have demonstrated that paraosteal osteosarcomas with areas of dedifferentiation usually show intralesional radiolucencies on radiological images. 

**Figure 6:**
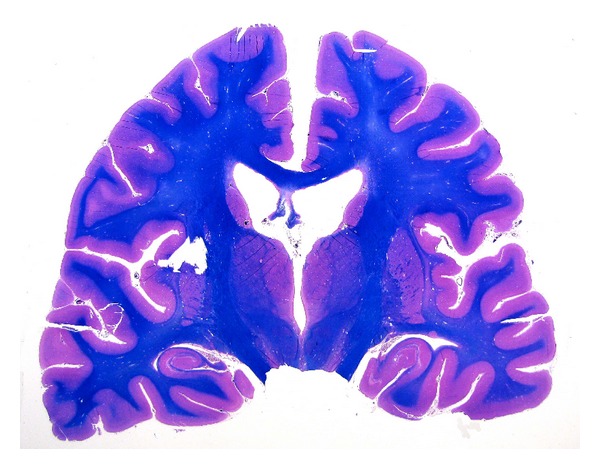
Large format section of brain, stained with the Luxol-fast blue technique, evidence the normal brain anatomical structures.

LS have also occasionally been used to study the inner ear anatomy [42], but have been largely used to study normal brain anatomy [43] and degenerative brain diseases. LS of brain tissue are stained using several histochemical methods that help to evidence grey and white matter (Figure 6) and the related lesions and, as recently demonstrated by Howell et al. [44], are useful to perform a detailed mapping of degenerative brain lesions. 

In practice, in the daily practice, LS are potentially useful to understand, diagnose, and manage the pathological lesions from all organs.

Accordingly, Slootweg and Grot [45] applied LS to stage the neoplastic lesions of the head and neck district, comprising the different areas of the oral cavity and the larynx. Tumours arising in the head and neck region often involve mandibular and maxillary bones and the surrounding soft tissues. Evaluation of the extent of the tumoural growth and tissue involvement, especially in this district, is of crucial importance to perform a correct staging. As tissues from head and neck region have different consistency. Slootweg and Grot [45] proposed to cut the surgical specimens using an engine driven water-cooled diamond saw and to obtain LS inclusive of bone and surrounding tissues. These LS are optimal for all types of neoplastic lesions affecting the head and neck region and can easily and unequivocally demonstrate the type and extension of tumoral growth. 

**Figure 7:**
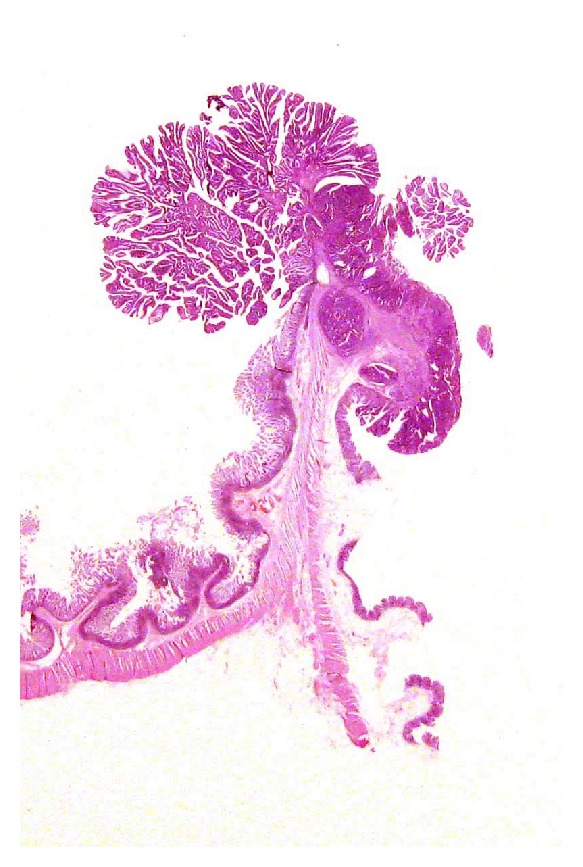
Large format section from an intestinal adenomatous polyp with adenocarcinoma. The level of invasion by the adenocarcinomatous area is easily assessed.

LS have been proven to be useful in visualizing gastrointestinal tumours [46] (Figure 7). During the last two decades, the wider use of colorectal endoscopies and the application of screening programs for colorectal cancer have led to the recognition of early neoplastic lesions that can be of difficult interpretation using conventional blocks histology. LS allow to visualize the whole lesions and to correlate the histological features with the endoscopic findings.

LS were useful to demonstrate the pathway of placental diffusion of cytomegalovirus infection in twins [47] and more recently to study the amyloid involvement in the heart [48].

**Figure 8:**
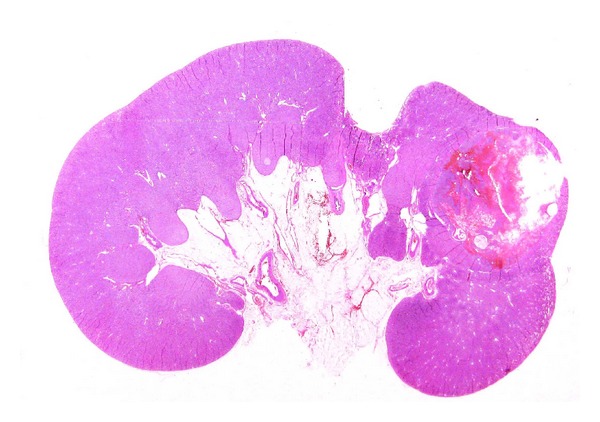
Example of large format section of a case of clear cell carcinoma of the kidney. The relationship with the tumour and the surrounding tissue, capsule, and urinary pelvis are well evident. In addition the renal parenchyma shows an area of pyelonephritis.

**Figure 9:**
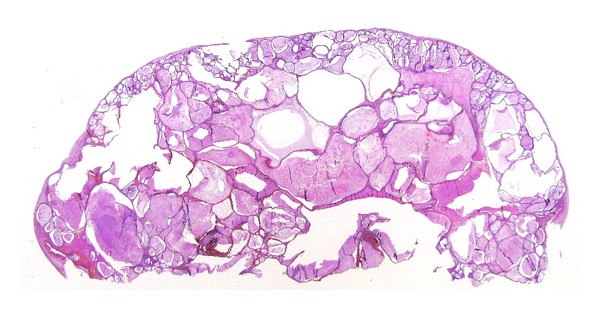
Mucinous cystoadenocarcinoma borderline of the ovary, measuring 11 cm in greatest axis. A whole section of the tumour leads to a more accurate diagnosis.

**Figure 10:**
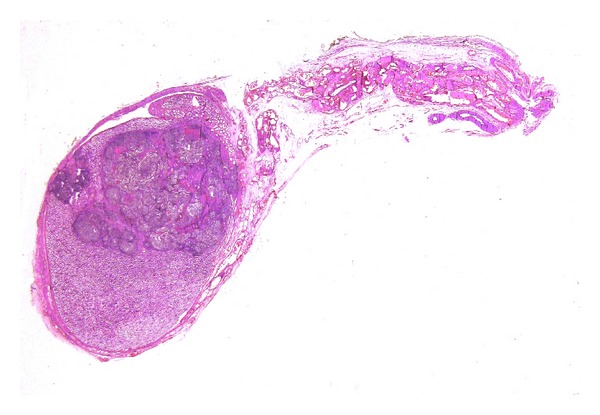
Testicular seminoma: the relationship between the tumour and the tunica albuginea is well evident.

For diagnostic purposes LS can be applied to almost organs (Figures 8, 9, and 10).

**Figure 11:**
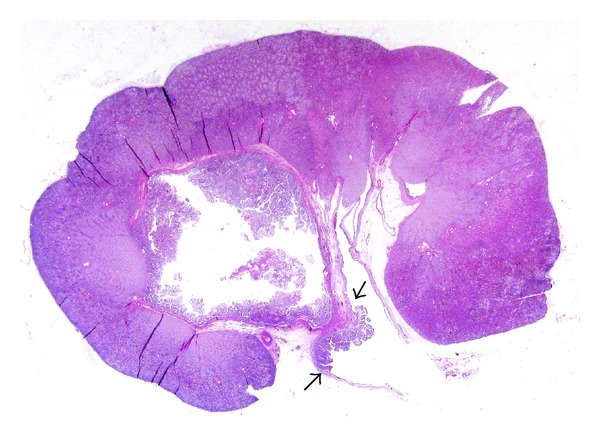
LS on a transitional cell carcinoma of the urinary pelvis demonstrates that the tumour shows two separate foci (arrows).

As shown in Figure 11 in a case of transitional cell carcinoma of the urinary pelvis, LS clearly demonstrated that the tumour under study was composed of two distinct neoplastic foci, separated by uninvolved urinary epithelium.

Currently in prostate pathology, LS constitute a standard of care in the staging of prostate cancer, as carefully explained by Montironi et al. [28] in this issue.

In addition as prostate cancer must be excluded in donor candidates during organ explanation, a method for cyrosectioning the whole prostate has been proposed [49, 50].

## 6. Conclusions

LS have been applied in pathology for research and diagnostic purposes since the beginning of the 20th century. In spite of this long history that downgrades the LS to the level of an “old” technique its value is still consistently useful in the every day practice, especially in tumour pathology both for breast and most organs. 

The criticism that the LS increase the cost and turnaround time of the surgical pathology routine work is not anymore tenable, as it has been demonstrated their cost-effectiveness and the turnaround time not longer than 24 hours. Therefore, an increasing application of LS in the daily pathological practice is auspicial.
